# Tuberculoma Masquerading as a Sixth Cranial Nerve Palsy in a Young Patient: A Case Report

**DOI:** 10.7759/cureus.59469

**Published:** 2024-05-01

**Authors:** Rinkle Gemnani, Keyur Saboo, Anil Wanjari, Sunil Kumar, Sourya Acharya

**Affiliations:** 1 Department of Medicine, Jawaharlal Nehru Medical College, Datta Meghe Institute of Higher Education and Research, Wardha, IND

**Keywords:** antitubercular therapy, case report, diplopia, sixth nerve palsy, tuberculoma

## Abstract

Tuberculosis is a worldwide health concern with a wide range of clinical manifestations. Rarely, it can involve the central nervous system in the form of tuberculomas. Although cranial nerve palsies or localized neurological signs are the most frequent presentations of tuberculoma, isolated sixth nerve palsy is unusual and rare. We report the case of a 17-year-old female who presented with isolated sixth nerve palsy, an unusual early manifestation of intracranial tuberculoma. We established the diagnosis through clinical, radiological, and laboratory evaluations and successfully managed the patient with antitubercular therapy. This case highlights the importance of considering tuberculoma as a differential diagnosis in cases of isolated cranial nerve palsies, especially in regions with a high prevalence of tuberculosis.

## Introduction

*Mycobacterium tuberculosis*, which causes tuberculosis, continues to be a major global health concern that affects millions of people globally. The central nervous system (CNS) is one of the many organs that can be affected by tuberculosis, despite the disease being most commonly known as a pulmonary infection [1,2].

There are two stages of CNS tuberculosis development. In the CNS, first, tiny tuberculous lesions appear as Rich’s foci. This occurs either during the bacteremia phase of the initial tuberculous infection or soon after. These early tuberculous lesions might be found in the meninges, the sub-pial or sub-ependymal surface of the brain, or the spinal cord. For years after the initial infection, these early lesions could stay dormant. The development of several types of CNS tuberculosis may result from the rupture or growth of these lesions in the future [1]. Tuberculomas are uncommon intracranial symptoms of tuberculosis that are defined as granulomatous lesions inside the CNS. The neurological manifestations of tuberculomas can range from typical symptoms, such as headache and seizures, to unusual presentations, such as localized neurological abnormalities [3,4]. However, isolated cranial nerve palsies as the early presentation of intracranial tuberculoma are exceedingly rare and pose diagnostic challenges.

This report documents the case of a 17-year-old female who presented with isolated sixth nerve palsy, an unusual and unique early manifestation of an intracranial tuberculoma. We aim to provide a detailed account of the clinical, radiological, and laboratory findings that led to this patient’s diagnosis and subsequent management. This case serves as a valuable illustration of the importance of considering tuberculoma within the differential diagnosis of isolated cranial nerve palsies, particularly in regions with a high prevalence of tuberculosis.

## Case presentation

A 17-year-old female visited our outpatient department with abrupt-onset horizontal diplopia associated with difficulty in moving her left eye outward for the past six hours. The patient gave a history of low-grade fever and left-sided headache for about 15-20 days. Double vision was evident when looking horizontally to the left. There was no drooping of the eyelids, no daytime or night-time fluctuation of the symptoms, and no accompanying discomfort or numbness around the orbit. She had no history of eye injuries, ear pain, ear discharge, unusual sensations around the face, or unusual facial movements. No history of drug use, diabetes mellitus, systemic hypertension, pulmonary or extrapulmonary tuberculosis, seizures, or dental procedures was reported. A thorough history was recorded which revealed no signs of ataxia, disorientation, weakness, vertigo, or dysphagia. The patient had received her childhood vaccinations according to schedule, and her prior medical history was insignificant. There was no history of any other neurological disorder in the family.

**Figure 1:**
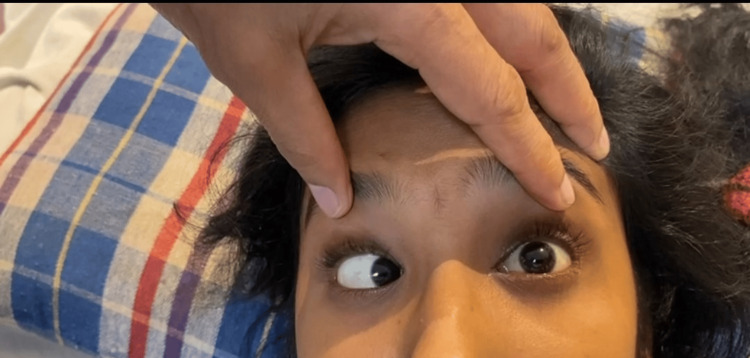
Image showing failure of abduction in the left eye with normal adduction in the right eye.

**Figure 2:**
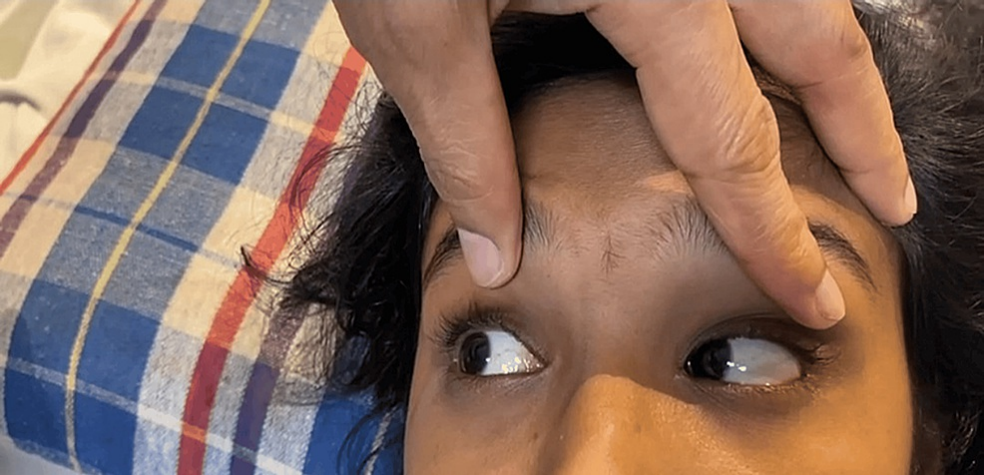
Image showing normal adduction in the left eye and normal abduction in the right eye.

The patient was conscious and oriented during the general physical examination. Her vitals were within normal limits. Anatomical components such as pupil size, reaction, eyelids, and fundus were normal on evaluation. Direct and consensual reflexes were found to be normal. In both eyes, the visual acuity was 6/6. On the finger confrontation test, there was a narrowing in the temporal eye field on the left side. On inspection, the left eye remained in the midline and was unable to move laterally, but the right eye moved medially in the normal fashion. The patient’s left eye could not be abducted but adduction was normal (Figures 1, 2). The systemic assessment of the cardiovascular, respiratory, and rest of the nervous system was normal.

On the day of the admission, her blood workup revealed raised white blood cell (22,500/mm^3^), normal hemoglobin, and platelet count with a raised erythrocyte sedimentation rate of 47 mm/first hour. Her renal and liver function tests were within normal limits. Viral markers for human immunodeficiency virus, hepatitis B surface antigen, and hepatitis C virus were negative. The thyroid profile was normal. An eye fundus examination was performed which was normal. A lumbar puncture showed raised cerebrospinal fluid cells, especially lymphocytes, adenosine deaminase, and proteins, and low sugar values, as shown in Table 1. The chest X-ray and electrocardiograph of the patient were normal. A tuberculin skin test was performed and no induration was seen after 72 hours of the test.

**Table 1:** Laboratory parameters of the patient with reference ranges. CSF = cerebrospinal fluid

Lab parameters	Observed value	Normal range
Hemoglobin	12.4 g%	12–15 g%
Mean corpuscular volume	84 fL	83–101 fL
Total leukocyte count	22,500 cells/mm^3^ (high)	4,000–10,000 cells/mm^3^
Platelets	2.85 lakhs/mm^3^	1.5–4.1 lakhs/mm^3^
Serum urea	20 mg/dL	19–43 mg/dL
Serum creatinine	0.9 mg/dL	0.66–1.25 mg/dL
Serum sodium	138 mmol/L	137–145 mmol/L
Serum potassium	4.5 mmol/L	3.5–5.1 mmol/L
Alkaline phosphatase	82 U/L	38–126 U/L
Alanine Aminotransferase	30 U/L	<50 U/L
Aspartate Aminotransferase	32 U/L	17–59 U/L
Albumin	3.6 g/dL	3.5–5 g/dL
Total bilirubin	0.5 mg/dL	0.2–1.3 mg/dL
Erythrocyte sedimentation rate	47 mm/hour (high)	0–20 mm/hour
CSF protein	222 mg/dL (high)	15–45 mg/dL
CSF adenosine deaminase	50 IU/L (high)	10–25 IU/L
CSF glucose	22 mg/dL (low)	40–70 mg/dL
CSF total leukocyte count	400 cells/µL (high), lymphocytes: 70%, neutrophils: 30%	<5 cells/µL
CSF red blood cells	1/µL	<2/µL

**Figure 3:**
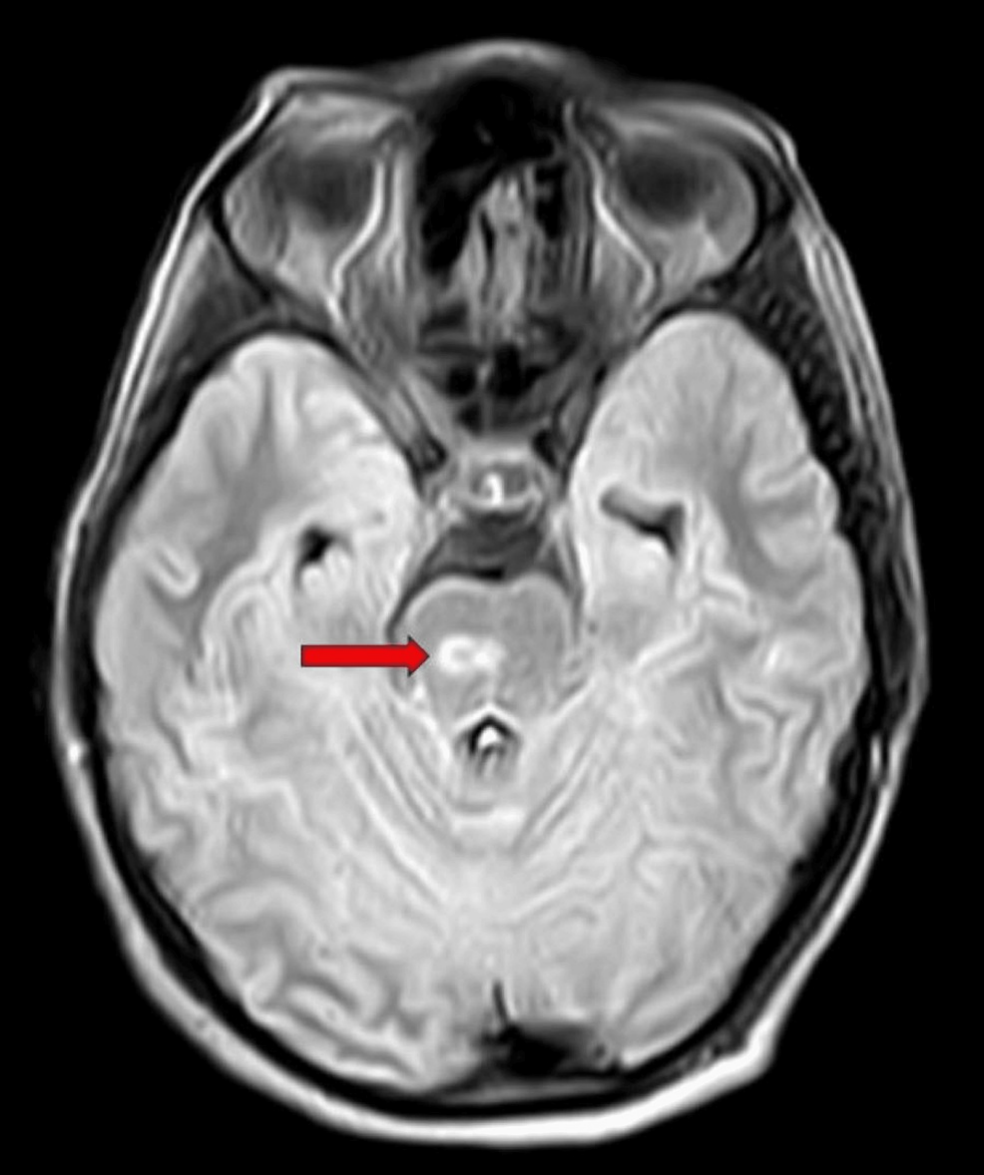
An axial section of magnetic resonance imaging showing a peripherally enhancing altered signal intensity lesion in the pons on the right side (red arrow).

**Figure 4:**
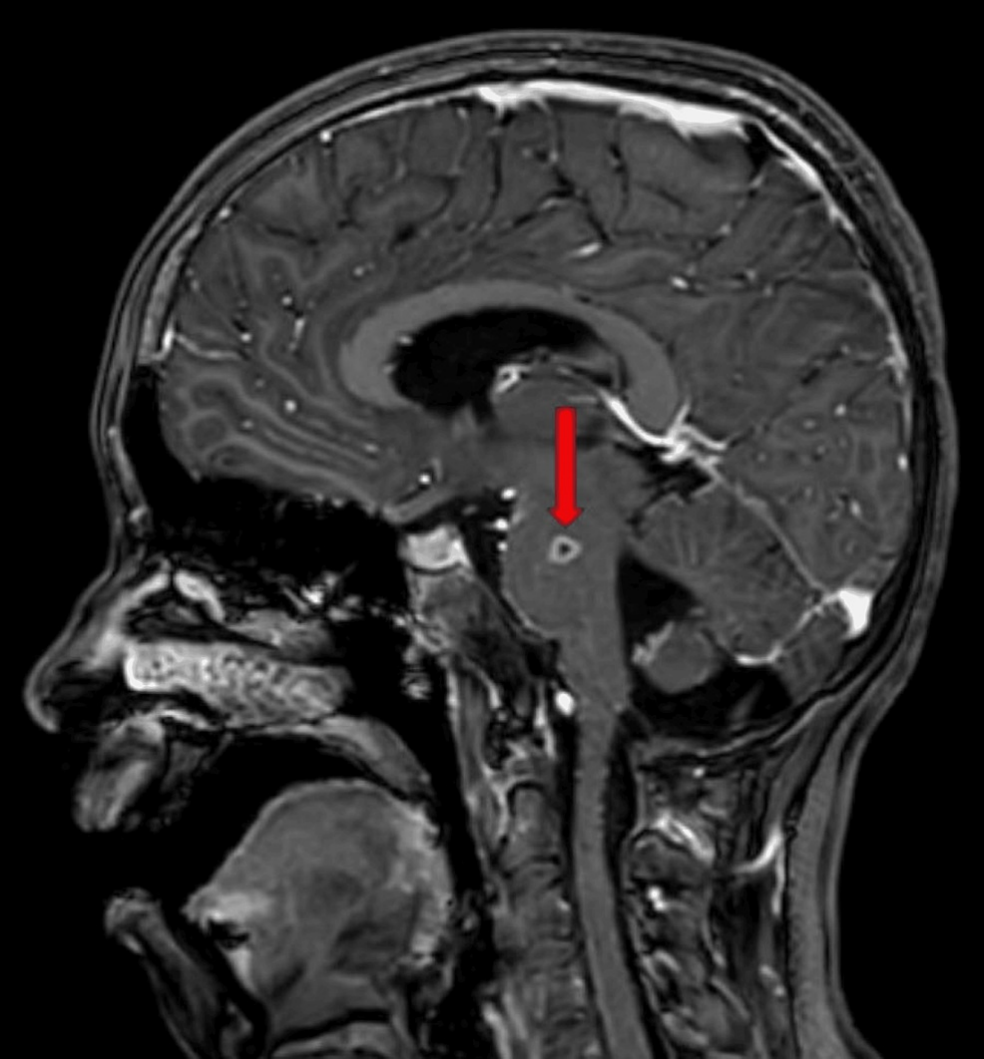
A sagittal section of magnetic resonance imaging showing a peripherally enhancing altered signal intensity lesion in the pons (red arrow).

A non-contrast magnetic resonance imaging scan of the brain revealed a peripherally enhancing altered signal intensity lesion in pons on the right side most likely tuberculoma (caseating granuloma with central liquefaction) (Figures 3, 4).

With signs of tuberculoma, the patient was put on antitubercular therapy with rifampicin, isoniazid, pyrazinamide, and ethambutol with dose according to her weight for two months, followed by a two-drug regimen (rifampicin and isoniazid) for 10 months, and corticosteroid therapy with tablet dexamethasone 8 mg thrice daily for the first six weeks. The patient was monitored for liver enzymes at the outpatient clinic. She improved symptomatically in six weeks, with a significant return of eye movements.

## Discussion

Tuberculosis remains one of the most common infectious disease-related causes of death. Tuberculosis of the CNS is the fifth most common and severe form of extrapulmonary tuberculosis. Intracranial tuberculomas may appear as solitary or multiple masses [5]. Solitary lesions can pose diagnostic challenges as they may resemble abscesses or tumors. Headaches, seizures, hydrocephalus, cranial nerve palsy, cerebral artery involvement, and localized ischemia are among the symptoms. However, the simultaneous involvement of multiple cranial nerves is common, isolated involvement of the abducent nerve is uncommon, and it can be an unusual yet early sign of intracranial tuberculoma, as seen in this case [6,7].

The abducens nerve has a longer peripheral route; hence, solitary abducens nerve palsy is very common. The nucleus of the abducens nerve is located in the caudal pons, close to the facial colliculus. Because the loop of the facial nerve and pontine gaze center are close to the abducens nerve nucleus, isolated nuclear lesions of the abducens nerve are relatively uncommon. The abducens nerve emerges from the brainstem near the pons and medullary pyramids. Because the nerve can be affected by any part along its lengthy intracranial course, a differential diagnosis requires considerable research to determine the underlying cause [1,8].

There could be various etiologies of isolated abducent nerve injury, including vascular, neoplastic lesions, degenerative causes, viral infections, inflammation, or trauma. In patients from developing countries, or in a patient with a history of tuberculosis exposure, intracranial tuberculoma should be considered as a possible differential diagnosis. Brainstem tuberculoma typically manifests with low-grade fever, weight loss, headache, vomiting, sixth and seventh cranial nerve affections, and unilateral motor and sensory complaints. Lesions along the extra-axial course of the nerve can cause isolated abducens nerve palsy [9]. The laboratory reports indicate an elevated erythrocyte sedimentation rate. Radiological imaging shows a nodular lesion with enhancement, consistent with an active inflammatory granuloma, suggestive of tuberculoma [6,10]. In our patient, the biopsy of the lesion was not performed due to suspected tuberculosis. During follow-up, the patient’s condition steadily improved with antitubercular medication. In our case, the isolated involvement of the abducens nerve by the tuberculoma in a young female patient resolved with treatment, which is rare.

## Conclusions

Isolated sixth cranial nerve palsy observed in our patient, as an initial presentation of tuberculoma, is exceptionally rare. Although diagnosing the disease is challenging and often delayed, a thorough exploration of extracranial sites for tuberculosis can lead to earlier and safer histopathologic confirmation. Antitubercular therapy and steroids are effective, but long-term clinical and radiological follow-up is crucial for good outcomes. Clinicians must maintain a heightened level of suspicion for unconventional presentations of tuberculosis, even among young patients devoid of classic symptoms. This case exemplifies the remarkable potential for resolution and improvement following appropriate therapeutic interventions in intracranial tuberculomas.

## References

[1] Kantharia S, Kantharia RA, Reddy DP (2021). Isolated 6th cranial nerve palsy: a rare manifestation of tuberculosis. Int J Otorhinolaryngol Head Neck Surg.

[2] Kakde YK, Talwar D, Bagga CS, Mahajan JN, Kumar S (2022). Scrofula presenting as tubercular meningitis: a neglected sequelae. J Clin Diagn Res.

[3] Jain D, Aggarwal HK, Rao A, Dahiya S (2017). Tuberculoma presenting as isolated sixth nerve palsy: a rare case report. Eur J Gen Med.

[4] Acharya S (2014). Pott’s spine with simultaneous neurotuberculosis - a rare case report. Case Study Case Rep.

[5] Agrawal A (2011). Paradoxical manifestation of cerebellar tuberculoma. Infect Dis Clin Pract.

[6] Perez-Malagon CD, Barrera-Rodriguez R, Lopez-Gonzalez MA, Alva-Lopez LF (2021). Diagnostic and neurological overview of brain tuberculomas: a review of literature. Cureus.

[7] Chaudhari P, Sawant R, Bedi GN, Desale R, Kumar S, Acharya S (2024). Case report on Mycobacterium tuberculosis presenting as Lemierre's syndrome: a reemerging catastrophe. Cureus.

[8] Smith DE, Blasi A (2009). Acquired abducens nerve palsy secondary to tuberculosis. Optometry.

[9] Abdolhoseinpour H, Abolghasemi S, Jangholi E, Naghi Tehrani KH (2018). Isolated oculomotor and abducens nerve palsies as initial presentation of cavernous sinus tuberculoma: case report and literature review. World Neurosurg.

[10] Acharya S, Shukla S, Goyal A, Irshad VS, Bagga C (2022). Disseminated tuberculosis with neurotuberculosis presenting as status epilepticus: a case report. J Clin Diagn Res.

